# Level of Asthenia and Life Quality among Students during the COVID-19 Second Wave in the Russian Federation

**DOI:** 10.1192/j.eurpsy.2025.1868

**Published:** 2025-08-26

**Authors:** V. I. Rozhdestvenskiy, V. V. Titova, I. A. Gorkovaya, Y. S. Aleksandrovich, D. O. Ivanov

**Affiliations:** 1 Department of Psychosomatics and Psychotherapy, Saint Petersburg State Pediatric Medical University, Saint Petersburg, Russian Federation

## Abstract

**Introduction:**

The COVID-19 pandemic significantly impacted the population in Russia, particularly students, who were forced to radically restructure their daily lives rhythm due to the introduction of distance learning, more homework, increasing the share of self-education. These factors could provoke the emergence of a particular psychopathological syndrome — asthenia, which is characterised by general and mental weakness, increased exhaustion, irritability, decreased productivity of cognitive processes, decreased motivation, sleep disorders and other psychosomatic disorders. These symptoms, in turn, could be associated with changes in the student’s quality of life.

**Objectives:**

The study aimed to assess the level of asthenia among students in humanities programs and to examine the relationship between asthenia indicators and quality of life.

**Methods:**

Data collection was conducted from January to April 2021 through a specially developed Google form. Thirty-five students at the universities of the Russian Federation took part in the study. We used MFI-20 to assess the level of asthenia, and WHOQOL-BREF to study the life quality. Both questionnaires were adapted for Russian-speaking respondents.

**Results:**

We found that 31% of respondents had symptoms of general asthenia, 29% had symptoms of decreased activity, 26% had symptoms of decreased motivation, 71% had symptoms of physical asthenia, and 26% had symptoms of mental asthenia. No statistically significant correlations were found between asthenia manifestations and life quality indicators. However, significant direct correlations were obtained between all quality-of-life domains, except for “microsocial support” and “social well-being”.

**Conclusions:**

In the conditions of the COVID-19 second wave, most students in Russia demonstrated physical asthenia symptoms. Asthenia manifestations were not related to the student’s life quality. Students in the pandemic conditions should observe the sleep, rest regime and optimal physical activity. Among students in Russia, the microsocial aspect of life quality (relations with partners, sexual life, relations with friends) are fairly detached from the broader social context (e.g. everyday life safety, healthy physical environment, and so on).

**Disclosure of Interest:**

None Declared

